# A simplified and efficient method for isolating small extracellular vesicles for comparative and comprehensive translational research

**DOI:** 10.1038/s41598-025-99822-y

**Published:** 2025-05-11

**Authors:** Prahalad Singh Bharti, Komal Rani, Rishabh Singh, Sanskriti Rai, Simran Rastogi, Manya Batra, Abhay Mishra, Sadaqa Zehra, Priya Kumari Gorai, Manda Venkata Sasidhar, Gyan Prakash Modi, Garima Malik, Neerja Rani, Kapil Dev, Thota Jagadeshwar Reddy, Krishna Kishore Inampudi, Fredrik Nikolajeff, Saroj Kumar

**Affiliations:** 1 Department of Biophysics, All India Institute of Medical Sciences, New Delhi, Delhi 110029 India; 2 Department of Pathology & Laboratory Medicine, All India Institute of Medical Sciences Bibinagar, Hyderabad, Telangana 508126 India; 3 Department of Anatomy, All India Institute of Medical Sciences, New Delhi, Delhi 110029 India; 4 Apollo Hospitals Educational and Research Foundation, Hyderabad, Telangana 500033 India; 5 Department of Pharmaceutical Engineering & Technology, Indian Institute of Technology BHU, Varanasi, Uttar Pradesh 221005 India; 6 Indian Council of Medical Research, New Delhi, 110029 India; 7 Department of Biotechnology, Jamia Millia Islamia, New Delhi, 110025 India; 8 Analytical Department, CSIR-Indian Institute of Chemical Technology, Hyderabad, Telangana 500007 India; 9 Department of Health, Education and Technology, Lulea University of Technology, 97187 Lulea, Sweden

**Keywords:** Small extracellular vesicles, sEVs isolation, Nanoparticle tracking analysis, Human saliva, Human plasma, Cell culture media., Biological techniques, Cell biology, Molecular biology, Biomarkers, Diseases

## Abstract

Small extracellular vesicles (sEVs) can provide information about the pathophysiology of the cells; therefore, sEVs have attracted considerable interest as possible diagnostic biomarkers. A key challenge lies in the necessity for simple and cost-effective sEV isolation methods to achieve high purity and yield suitable for research and clinical applications. We are introducing a comprehensive study on isolating sEVs using a novel cocktail strategy that integrates chemical precipitation and ultrafiltration with a two-step filtering process to ensure a highly pure and homogeneous population and further compared with PEG-based precipitation, ultra-centrifugation, and size-exclusion-chromatography columns. The isolated sEVs from each protocol are quantified for size and yield using nanoparticle tracking analysis, morphologically characterized through transmission electron microscopy, and validated by quantifying the expression profiles of sEV surface biomarkers. Furthermore, the study explores the applicability of our method for downstream multi-omics analyses. The results highlight the efficacy of the proposed protocol, demonstrating the ease and efficiency of isolating sEVs from different biofluids with minimal laboratory requirements and confirming the compatibility with multi-omics analyses. These findings position our method as particularly valuable for translational research, offering a promising avenue for advancing the study and application of sEVs in diagnostic and therapeutic research.

**Supplementary Information:**

The online version contains supplementary material available at 10.1038/s41598-025-99822-y.

## Introduction

The phrase “extracellular vesicle” (EV) refers to any naturally occurring particle released from a cell that is bounded by a lipid bilayer and is incapable of replicating, meaning it lacks a functioning nucleus^1,2^. Extracellular vesicles (EVs) can be categorized into two distinct subtypes: ectosomes, which possess a size range of 100 to 1000 nanometers and are released by cells through the process of cytoplasmic membrane budding. The second subtype is small extracellular vesicles (sEVs), characterized by a size range of 30 to 200 nanometers, commonly referred to as exosomes, which are released through the fusion of multivesicular bodies (MVBs) with the plasma membrane of the cell. sEVs have emerged as invaluable tools in diverse domains of biomedical research and clinical applications owing to their facilitation of intercellular communication, as they transport cargo from their parent cells to the extracellular space of recipient cells. Extensive research is currently being conducted to elucidate the role of sEVs in diagnostics, therapeutics, drug delivery, and related fields. The primary obstacle resides in the establishment of standardized methodologies for optimal isolation techniques to fully exploit their therapeutic and diagnostic capabilities. Significant advancements in developing novel and improved techniques for isolating sEVs from different biological fluids, including blood, urine, saliva, and cerebrospinal fluid, have been made over the past two decades^3–5^. Based on current research, sEV can be called a non-invasive fluid and is used in many research fields, such as biomarker discovery, sEV therapy, drug delivery, etc^6,7^. However, challenges persist in working with sEVs; the biggest issue is isolating highly purified sEVs with minimal infrastructure. The most commonly used methods for the isolation of sEVs from various sources include size-exclusion chromatography, ultra-centrifugation, and immunoaffinity capture^8^. Ultracentrifugation (UC) is the most commonly used method for sEVs isolation. This method involves several rounds of centrifugation at high speeds, which separate sEVs from other cellular particles based on their density and size. Despite its effectiveness, ultracentrifugation is time-consuming and requires specialized equipment and technical expertise^5^. Size-exclusion chromatography (SEC) separates sEVs based on size and hydrodynamic properties using a porous column that allows smaller molecules to enter the pores while larger particles are eluted first. Although SEC is faster and more efficient than ultracentrifugation, it may be less effective in isolating sEVs from complex biological fluids^4,5^. Immunoaffinity capture, a more recent method, captures sEVs using antibodies specific to exosomal surface markers like CD63, CD81, or CD9. This method is highly specific and can isolate sEVs from complex fluids, but it requires specific antibodies and can be expensive^9^.

In addition to these methods, new techniques such as microfluidics, acoustic separation, and precipitation-based methods have also been developed. These methods offer faster and more efficient isolation of sEVs with high purity and yield^9,10^. Another challenge is characterizing the isolated sEVs, assessed by three parameters: (1) ultrastructural morphology using transmission electron microscopy (TEM), (2) quantification and size of sEVs by nanoparticle tracking analysis (NTA), and (3) expression of sEV protein markers by western blot analysis^11,12^. While all these parameters can be used in research studies, employing all three in a clinical setting is impractical. Additionally, only some methods are universally applicable, confusing researchers when selecting the appropriate protocol for their studies. Hence, easy and inexpensive ways are desperately needed to attain high purity and yield that is feasible for research and clinical setups with minimum facility requirements.

## Results

We used volume normalization for all investigations because data indicate that isolated sEVs constitute a diverse population in clinical samples. All three samples (cell culture, plasma, and saliva) had the same volume at the start of the experiment from every person to separate sEVs.

### Ultrastructural morphology of sEVs

**Fig. 1:**
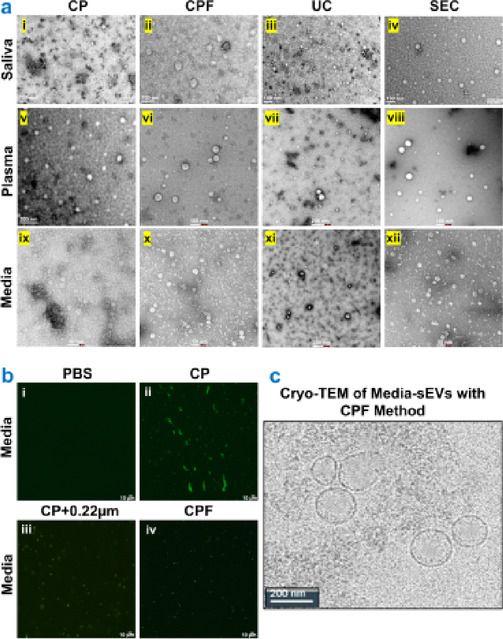
Transmission electron microscopy (TEM) and laser confocal microscopy of sEVs. (**a**) Transmission electron microscopy of sEVs isolated from biological samples using different methods. sEVs isolated with PEG-based precipitation (CP) (**a – i**,** v**,** ix**); PEG-based precipitation with ultrafiltration (CPF) (**a – ii**,** vi**,** x**); Ultracentrifugation (UC) (**a – iii**,** vii**,** xi**); and Size exclusion chromatography (SEC) columns (**a – iv**,** viii**,** xii**) from saliva (**a – i to iv**), plasma (**a – v to viii**) and conditioned cell culture media (**a – ix to xii**). (**b**) CD9 expression in media isolated sEVs. (**b-i**) PBS control, (**b-ii**) sEVs isolated with CP method, (**b-iii**) sEVs isolated with CP method followed by 0.22 μm syringe filter, (**b-iv**) sEVs isolated with CPF method (Scale bar is 5 μm). (**c**) Cryo-TEM of media-derived sEVs, isolated by CDF methods.

Transmission electron microscopy (TEM) was performed to characterize the ultrastructure of the isolated sEVs from cell culture media, plasma, and saliva (Fig. 1a). TEM micrographs revealed that the sEVs were round vesicular structures with a 30–150 nm diameter range. Some of the sEVs contained internal vesicles, and all of the sEVs had a lipid bilayer membrane. These results suggest that the sEVs were isolated from all three biological samples using the abovementioned methods. The purest sEVs were isolated from the PEG-precipitation with the ultrafiltration (CPF) method with no to very few non-vesicular particles or artifacts (Fig. 1a – ii, vi, x) in comparison to other methods, like the CP method (Fig. 1a – i, v, ix), and UC method (Fig. 1a – iii, vii, xi). However, less sEV concentration was observed in the SEC method (Fig. 1a – iv, viii, xii). sEVs isolated from cell culture media (Fig. 1a – ix to xii) are smaller than saliva and plasma-derived sEVs. Furthermore, sEV validation was performed using an anti-CD9 antibody, a surface marker for sEVs, immunostaining and observed under a laser confocal microscope^13^. The sEVs isolated with the CPF method were observed with highly specific CD9 expressions in comparison to the CP method (Fig. 1b – i to iv). Also, the sEVs isolated with CP followed by 0.22 μm syringe filtration showed more specified CD9 expression than the CP-only method but less specific staining with the CPF method (Fig. 1b). Combining both transmission electron microscopy and laser confocal microscopy, we were able to ascertain the purity of sEVs isolated with the CPF method. Cryo-TEM was also performed for media-derived sEVs isolated with the CPF method, showing double-membraned spherical vesicles with a size of 100–200 nm (Fig. 1c).

## Quantification of sEVs

**Fig. 2:**
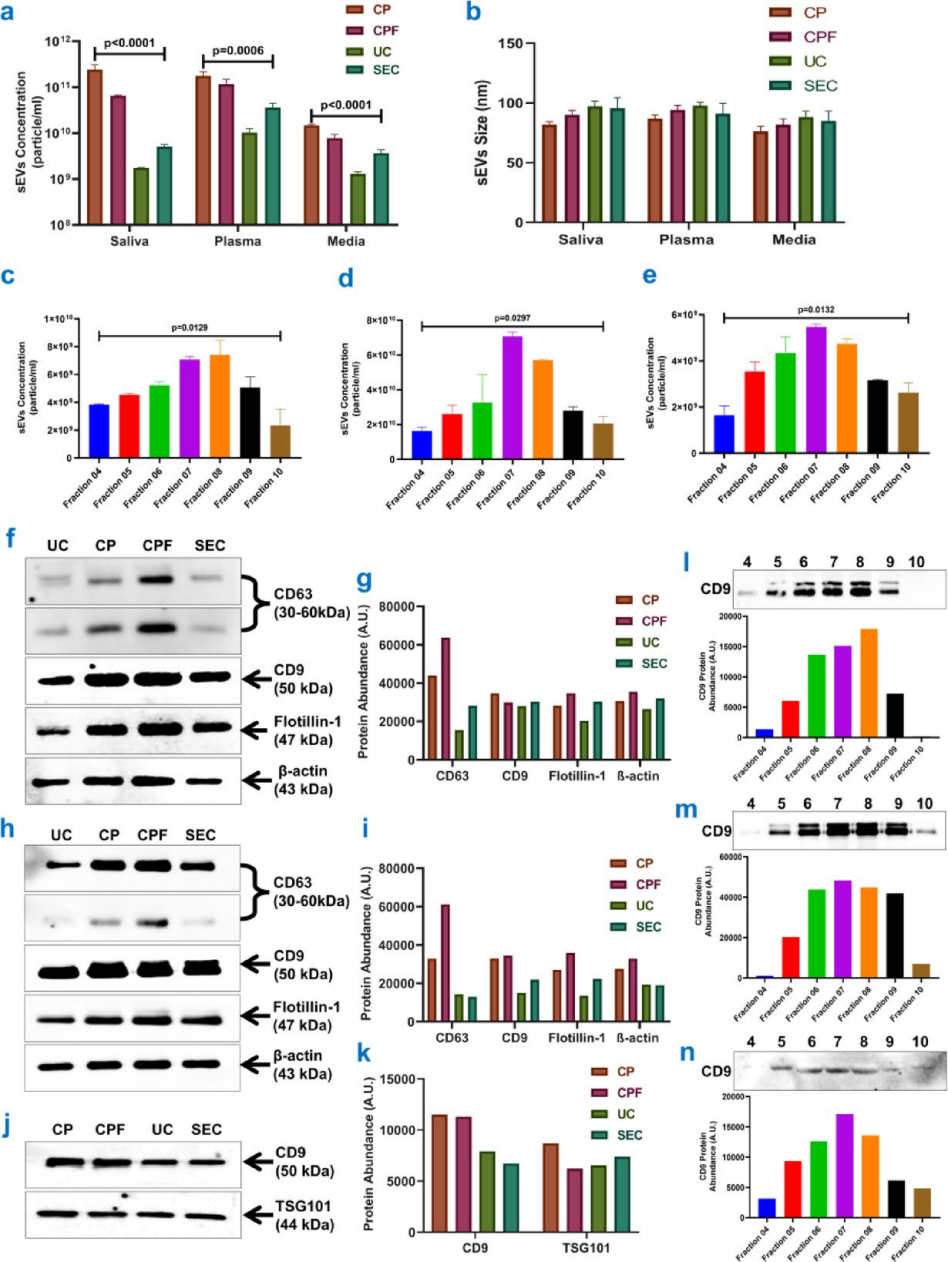
Concentrations & expression profiles of sEVs isolated from saliva, plasma, and conditioned cell culture media. sEVs concentrations (**a**) and size distribution profile (**b**) in different samples were isolated using different methods: PEG-based precipitation (CP), PEG-based precipitation with ultrafiltration (CPF), ultracentrifugation (UC), and size exclusion chromatography (SEC) column. sEVs concentrations in different fractions of SEC method in saliva (**c**), plasma (**d**), and media (**e**) (Mean ± SEM). The expression profiles of sEVs markers (CD63, CD9, Flotillin-1, & TSG-101) in the saliva-derived sEVs and their densitometric analysis (**f**,**g**), the plasma-derived sEVs and their densitometric analysis (**h**,** i**), and the conditioned cell culture media-derived sEVs and their densitometric analysis (**j**,** k**). β-actin is a loading control. CD9 expressions in different fractions of sEVs isolated using the SEC method from saliva (**l**), plasma (**m**), and conditioned cell culture media (**n**) with their respective densitometric analyses. The sEVs isolation methods are precipitation (CP), precipitation with ultrafiltration (CPF), ultracentrifugation (UC), and size exclusion chromatography (SEC) columns. (Kruskal Wallis post-hoc analysis in **a-e**).

sEVs were quantified for concentrations and size distribution using nanoparticle tracking analysis (NTA) in scatter mode from saliva, plasma, and cell culture media. The samples were prepared using all four methods: PEG-based precipitation (CP), CPF, UC and SEC column. The highest concentration of sEVs in all samples was isolated by the CP method with 2.43E + 11 particles per ml in saliva, 1.76E + 11 in plasma, and 1.46E + 10 in cell culture media. In contrast, the lowest concentrations were isolated from UC methods with 1.74E + 09 in saliva, 1.02E + 10 in plasma, and 1.3E + 09 in media (Fig. 2a, Supplementary Table S2).

The sEVs isolated using the CPF and SEC methods were successively lower in concentration than the CP method but higher than the UC method (Fig. 2a, Supplementary Table S2). Also, the fraction-wise sEV concentration distribution of the SEC method from all samples represents that different samples show different fraction-wise concentration distributions using the same protocol (Fig. 2c–e), highlighting the requirement of optimization for different sample types while using the same size exclusion columns. Further, the NTA analysis revealed a size distribution with the biggest sEVs isolated with the UC & SEC methods, 97.13 ± 4.3 nm and 95.5 ± 8.9 nm from saliva, and 88.13 ± 5.1 nm and 84.97 ± 8.2 nm from conditioned cell culture media, respectively; whereas, in plasma samples, the biggest sEVs were isolated with UC and CPF methods, 97.83 ± 2.8 nm and 94.13 ± 4.6 nm, respectively. The smallest sEVs were isolated with the CP method, 81.87 ± 2.5 nm, 86.9 ± 3.9 nm, and 76.13 ± 4.4 nm in saliva, plasma, and conditioned cell culture media, respectively (Fig. 2b, Supplementary Table S2). Also, the variation in size distribution is highest in the SEC method from all samples, owing to the fraction collection procedure, showing a more heterogeneous population of sEVs isolated with the SEC method than other methods. Additionally, we calculated the particle-to-protein ratio and found that the UC and SEC methods had higher ratios than the CP and CPF methods (Supplementary Figure S7). The CPF method exhibited a higher particle-to-protein ratio than the CP method. These results indicate that when assessing purity using this ratio, the UC and SEC methods achieved the highest purity, followed by the CPF method. In contrast, the CP method showed the lowest sEV purity.

## Expressions of sEVs protein markers

The sEVs were isolated from saliva, plasma, and conditioned cell culture media using previously reported methods. The protein content of the samples was determined using the BCA method (Supplementary Figure S6). In Western blot analysis, primary antibodies against CD63, CD9, and Flotillin-1 were utilized, followed by secondary antibodies conjugated to horseradish peroxidase. The resulting Western blot exhibited robust CD63, CD9, and Flotillin-1 protein expression in the sEV samples. The CD63 multiple bands were observed between 30 and 60 kDa, whereas the CD9 band was observed around 50 kDa, the Flotillin-1 band was observed around 47 kDa and the TSG101 band was observed around 44 kDa (Fig. 2f, h, j, Supplementary Figures S1-S3), consistent with previous reports^14,15,16^. As a loading control, the band of β-actin was measured to be around 43 kDa. These results indicate that the isolated sEVs from saliva, plasma, and conditioned cell culture media express the expected sEV-associated proteins. The volume normalization approach is used in this study to load an equivalent volume of sEV samples, and densitometry analysis with ImageJ software was used to quantify the levels of protein expression.

Notably, the CPF method exhibited higher CD63 protein expression, suggesting the highest sEV yield in salivary (Fig. 2f, g, Supplementary Figures S1) and plasma-derived (Fig. 2h, i, Supplementary Figures S2) sEVs. Furthermore, the expression of CD9, Flotillin-1, and β-actin proteins was marginally higher in CPF than in CP and considerably higher than the other two techniques. These findings establish CPF as the optimal method for high sEV yields, with CP ranking as the second-best method (Fig. 2f-i). The expression of CD63 protein significantly increased in salivary and plasma-derived sEVs using the CPF method compared to other methods (Fig. 2f-i). However, the expression of CD9, Flotillin-1, and β-actin proteins remained relatively stable (Fig. 2f-i). In sEVs isolated from conditioned cell culture media, CD9 and TSG101 expressions were measured, with CD9 being higher in CP and CPF methods. TSG101 protein expression remained consistent across all methods (Fig. 2j, k, Supplementary Figure S3). Additionally, a Western blot analysis of sEVs isolated from all three types of samples using the SEC method against CD9 showed expression increasing from the 4th to the 10th fraction, peaking in the 7th or 8th fraction, depending on the sample type (Fig. 2l-n, Supplementary Figure S4). We also checked the expression of Apolipoprotein B, and observed a decrease in ApoB expression with the CPF method compared to the CP method, indicating reduced plasma protein contamination in our CPF method (Supplementary Figure S5). Among the three methods, the UC method exhibited the lowest ApoB expression in plasma-derived sEVs, suggesting the least protein contamination. These results were validated by sEV concentrations in the same fractions (Fig. 2c-e).

## Evaluation of plasma-derived sEVs for multi-omics

**Fig. 3:**
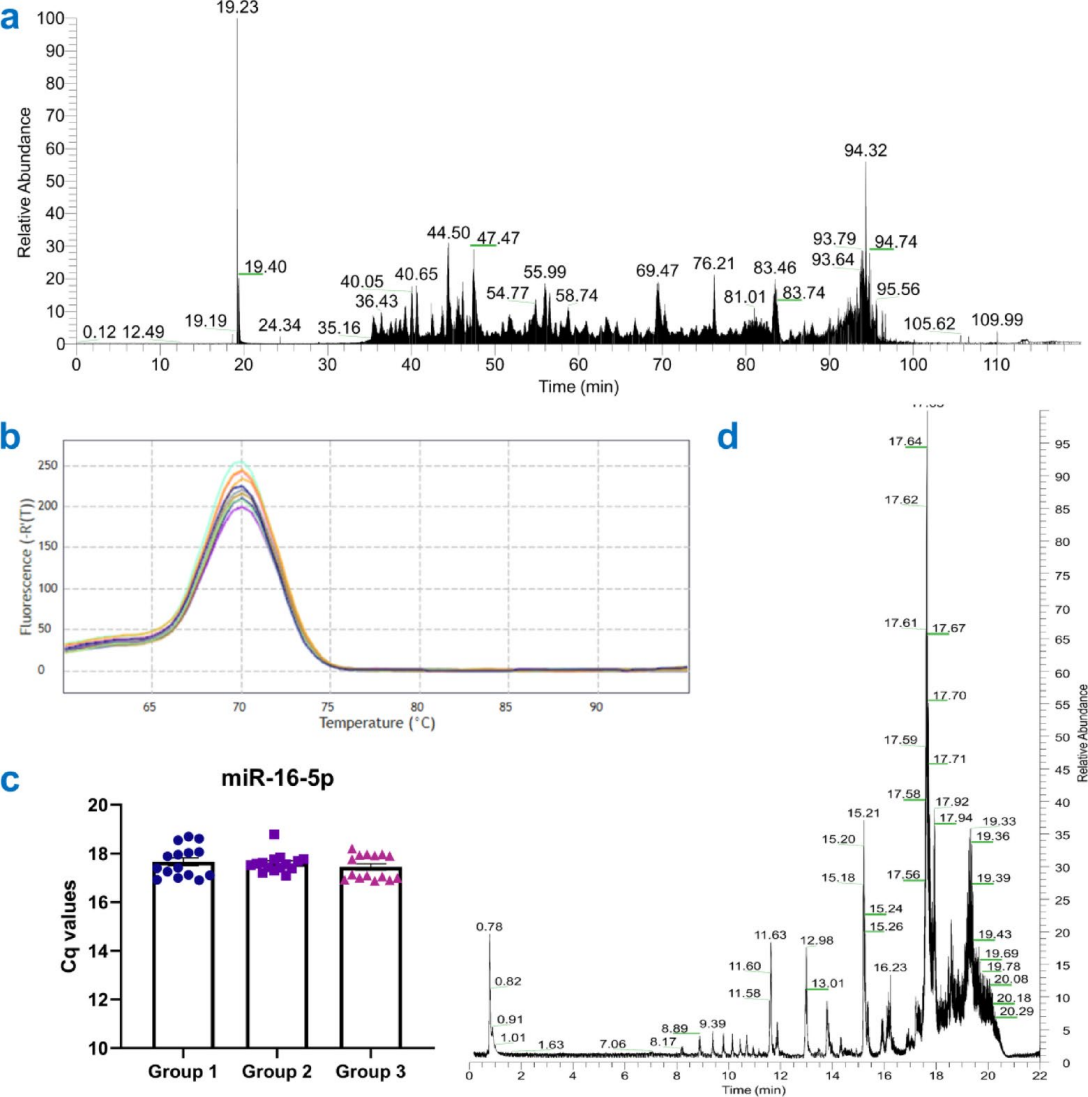
Multi-omics applications of plasma-derived sEVs isolated using precipitation with ultrafiltration (CPF) method. (**a**) A total ion liquid chromatography-mass spectrometry (LC-MS) chromatogram of sEVs proteins with relative abundance by retention time. (**b**) Melt-curve analysis of miR-16-5p reveals that single peaks were seen in the melting curves at such annealing temperatures, suggesting a highly selective amplification, and (**c**) raw Cq values for miR-16-5p in three samples. (**d**) A total ion LC-MS chromatogram of sEVs lipids with relative abundance by retention time.

The plasma-derived sEVs were processed for mass spectrometry analysis, and a total ion liquid chromatography-mass spectrometry (LC-MS) chromatogram with relative abundance against time was generated (Fig. 3a). This chromatogram derived from a two-hour Orbitrap LC-MS/MS run of tryptic digested sEV-derived proteins. Here, we have evaluated the compatibility of the CPF method with mass spectrometry analysis, and the total ion chromatogram shows separated and segregated peptide peaks comparable to other studies^17,18,19,20^, indicating efficient chromatographic separation, high sample complexity, and overall good quality.

Further, to show efficient isolation of plasma-derived sEVs with the CPF method, total RNA was isolated and processed for quality check for small RNA sequencing analysis. The quality of sEVs-derived RNA was checked using a small RNA target (miR-16-5p) because, since the plasma-derived sEV have a deficit of rRNA, the purified RNA from this source does not have prominent rRNA bands (as in Cellular RNA), which will lead to a very low RIN value. Therefore, the best way to evaluate the quality of the purified RNA is to amplify a highly abundant small RNA target. For this purpose, we have chosen miR-16-5p, which is reported to be a stable reference gene in sEVs^21–25^. In the melt-curve analysis of miR-16-5p, a single peak was observed that exemplifies the primer specificity as a single PCR product is formed, indicating a precise amplification (Fig. 3b). Also, the raw Cq values for miR-16-5p in all three samples are shown here (Fig. 3c), which illustrates that the reference miRNA is not only stably expressed throughout but also indicates higher initial copy numbers of the target, justified the quality of RNA isolated from plasma-derived sEVs with the CPF method^16,26,27^. Finally, to study the total lipid profile of plasma-derived sEVs with the CPF method, total lipid was isolated from sEVs with the Folch method and processed for liquid chromatography with tandem mass spectrometry, and a total ion chromatogram with relative abundance against time was generated (Fig. 3d) that depicts a total ion LC-MS chromatogram of Orbitrap LC-MS/MS run of 22 min of total lipid. Here, we have evaluated the compatibility of the CPF method with mass spectrometry analysis, and the total ion chromatogram demonstrates concordance with established studies^20,28,29^, affirming the CPF method as a robust approach for the comprehensive analysis of sEV lipid content.

## Discussion

The arena of extracellular vesicle (EV) research has witnessed remarkable growth over the past two decades, fuelled by the recognition of small extracellular vesicles (sEVs) as essential intermediaries of intercellular communication. This study delves into the challenges associated with sEV isolation and characterization, proposing a novel cocktail strategy and comparing it against conventional techniques. The study uses three diverse biofluids - saliva, plasma, and cell culture media - to isolate sEVs. It evaluates the efficacy of a protocol incorporating PEG-based precipitation and ultrafiltration methodologies with sequential centrifugation. This study highlights other established methods for sEV isolation, such as ultracentrifugation and (SEC), each with advantages and limitations. The time-consuming nature and specialized equipment required for ultracentrifugation and the potential inefficiency of SEC in complex biological fluids underline the need for alternative approaches. Still, the challenge remains in selecting a method that ensures high purity and yield while being easy and cost-effective, especially in research and clinical settings with minimal facility requirements. The study emphasizes the importance of selecting an effective isolation protocol for high-quality samples. The protocol developed aims to yield sEVs in their purest form with high yield, enhancing the reliability and integrity of downstream analyses. A comprehensive comparative analysis against conventional techniques (Table 1), including ultracentrifugation, PEG-based precipitation, and SEC, provides valuable insights into the efficacy of the proposed protocol. The ultrastructural morphology analysis using transmission electron microscopy (TEM) provides visual evidence of successful sEV isolation from all three biological samples using the new protocol. The PEG-precipitation with ultrafiltration (CPF) method stands out for producing highly pure sEVs with minimal non-vesicular particles or artifacts. Furthermore, quantifying sEVs using nanoparticle tracking analysis (NTA) reveals that the CPF method yields the highest concentration of sEVs in saliva, plasma, and cell culture media. Interestingly, this study highlights the importance of optimization for different sample types, as seen in the fraction-wise concentration distribution using SEC. These results are also confirmed with Western blot analysis, which further validates the efficacy of the CPF method by demonstrating higher expression of sEV-associated proteins compared to other methods. A comprehensive comparative analysis of sEVs characterization techniques outlines the advantages and disadvantages of each method (Table 2). This study goes beyond isolation and characterization, extending its evaluation to downstream applications like mass spectrometry analysis, small RNA sequencing, and lipid profiling. The compatibility of the CPF method with mass spectrometry is demonstrated through a total ion chromatogram, showcasing separated and segregated peptide peaks. The quality of sEV-derived RNA is affirmed by the stability of miR-16-5p expression, and the study successfully explores the total lipid profile of sEVs using the CPF method.

**Table 1:** Comparative analysis of sEV isolation methods.

Technique	Cost	Time (Sample Processing Time)	Purity	Yield	Recovery^6^	Clinical Applicability	Remarks
Ultra-centrifugation (UC)	Instrument Costly Reagents Cost-effective	Time-Consuming (5–6 h)	Very Good	Low	5–25%	Low	Large-scale preparation, Co-isolation of contaminants, Potential mechanical damage due to high-speed centrifugation
Size-Exclusion Chromatography Column (SEC)	Cost-effective (special columns)	Fast (4–5 h)	Better	Good	10–40%	Good	Different sizes of EVs in different fractions, mixed populations, and high pressure may shatter big vesicles into little ones
Chemical Precipitation (CP)	Cost-effective	Time-Consuming (11–13 h)	Good	High	≥ 30%	Good	General lab equipment, applicable to all sample amounts, co-separation of contaminants, impacting subsequent investigation and quantitation.
Chemical Precipitation + Ultrafiltration (CPF)	Cost-effective	Time-Consuming (11–13 h)	Better	Good	20–30%	High	General lab equipment, applicable to all sample amounts

**Table 2:** Comparative analysis of sEV characterization methods.

Techniques	Advantages	Disadvantages
Physical Characterization - Quantitation (Size and Concentration)		
Nanoparticle Tracking Analysis (NTA)	Size and concentration, Simple and fast	More suitable for tiny particles
Physical Characterization – Visualization by Microscopy		
Electron Microscopy (EM)	Best resolution, outer-surface topography & ultrastructure imaging can be done directly	Costly, Time-taking procedure, Expansive and low output
Laser Confocal Microscopy	Faster than other available techniques, Detection of sEV-specific proteins, Direct imaging	Costly, Time-taking procedure, No ultrastructure
Characterization		
Western Blotting and ELISA	Detection of sEV-specific proteins, Detection of protein size and quantity	Costly, Lesser Lower accuracy and performance, cross-reaction.
qRT-PCR	Less sample amount, higher resolution, higher output	Restricted to already identified RNA targets

**Fig. 4:**
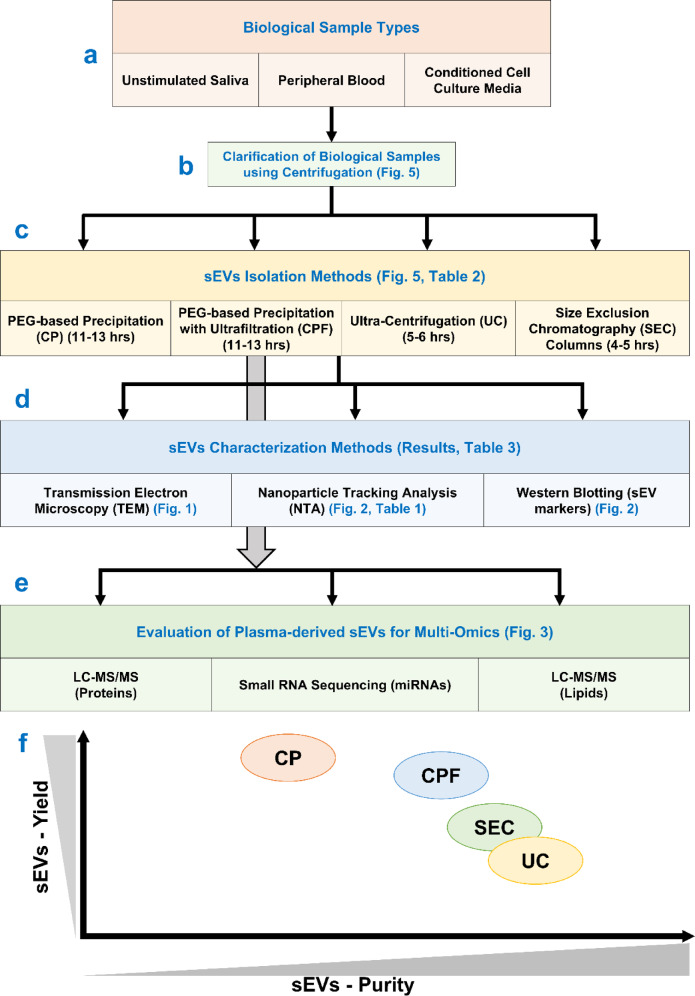
Schematic representation of the experimental design and study overview. (**a**) The types of biological samples used in this study are unstimulated saliva, peripheral blood, and conditioned cell culture media; (**b**) the clarification step for biological samples; (**c**) sEV isolation methods; (**d**) characterization methods for isolated sEVs; (**e**) downstream applications of plasma-derived sEVs isolated with the CPF method; and (**f**) comparison between different sEV isolation methods for biological samples.

Our study offers a comprehensive evaluation of small extracellular vesicle (sEV) isolation and characterization techniques across diverse biological samples, including saliva, plasma, and conditioned cell culture media. The implementation of volume normalization across all experiments ensured a consistent and standardized approach. Notably, the CPF (precipitation and filtration) method stood out for its efficiency in isolating sEVs with high purity and yield. Our findings demonstrate that the CPF method surpasses ultracentrifugation (UC) and size-exclusion chromatography (SEC) in terms of sEV yield and offers greater purity compared to PEG-based precipitation (CP) techniques (Fig. 4). We meticulously outline each protocol, emphasizing the crucial steps necessary for the efficient isolation and characterization of sEVs from biological samples. Our approach simplifies the process of isolating sEVs from various biofluids and conditioned cell culture media, requiring minimal laboratory resources. This balance of high yield, purity, and compatibility with downstream analyses makes our method particularly advantageous for a range of applications, from foundational research to clinical diagnostics. This study thus provides essential insights for refining sEV isolation protocols, significantly contributing to the fields of biomarker discovery and therapeutic development.

## Methods

### Experimental design and overview of the protocol

**Fig. 5:**
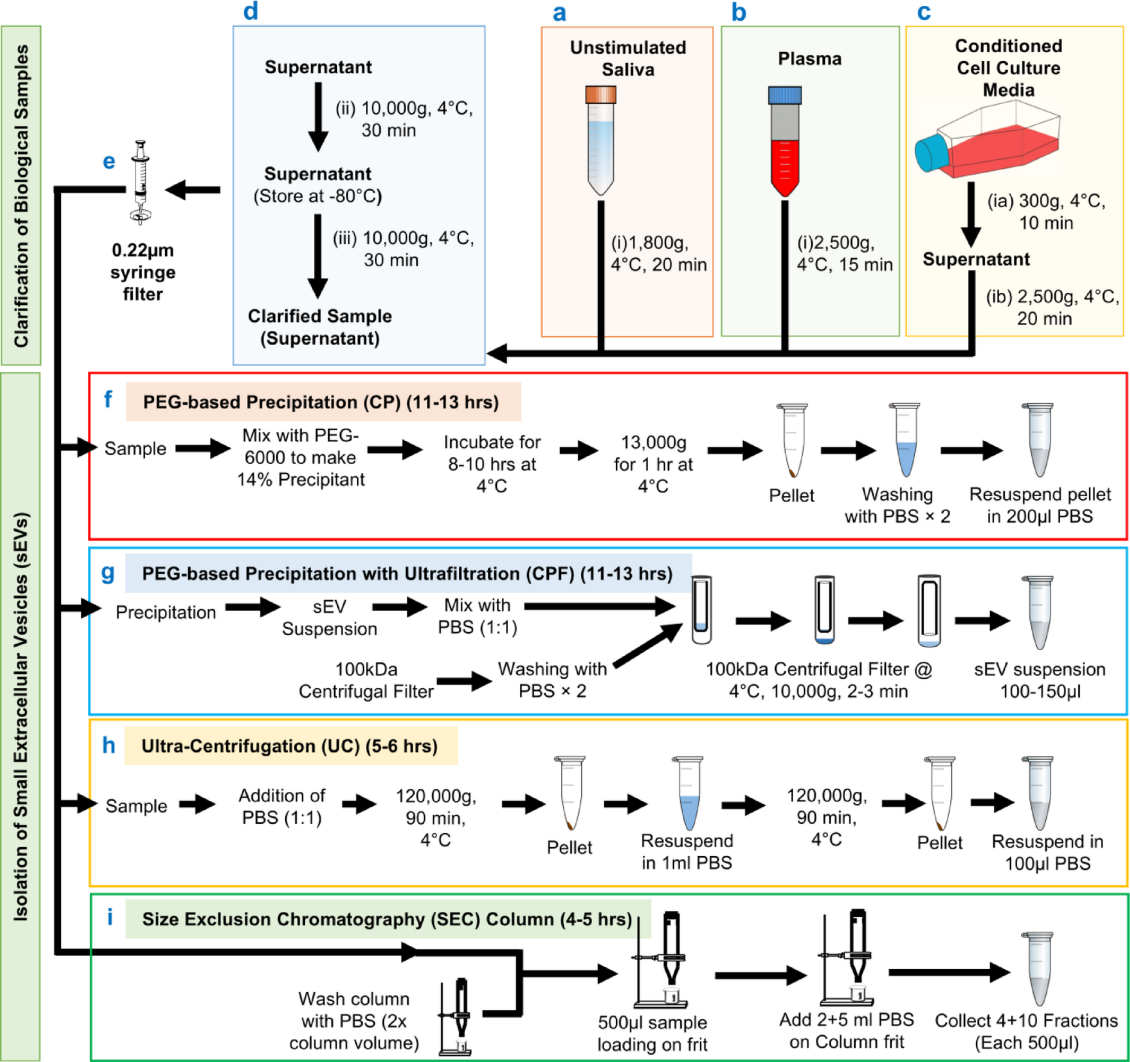
Clarification steps for biological samples and isolation of sEVs using four different protocols. (**a**) Unstimulated saliva; (**b**) plasma; (**c**) conditioned cell culture media samples before sEV isolation; (**d**) final clarification step; (**e**) filter with 0.22 μm syringe filters; (**f**) PEG-based precipitation (CP); (**g**) PEG-based precipitation with ultrafiltration (CPF); (**h**) Ultracentrifugation (UC); and (**i**) Size Exclusion Chromatography (SEC) columns.

In small extracellular vesicle (sEV) research, selecting an effective isolation protocol is pivotal to obtaining high-quality samples for comprehensive analysis. In this study, we have harnessed the potential of three diverse biofluids - saliva, plasma, and cell culture media - as sources for isolating sEVs. Our protocol incorporates PEG-based precipitation and ultrafiltration methodologies with a sequential centrifugation process (Figs. 4 and 5, Supplementary Table S1). The meticulous design of this protocol aims to yield sEVs in their purest form with high yield, enhancing the reliability and integrity of downstream analyses. A comparative assessment against conventional techniques, such as ultracentrifugation (UC)^3,17^, PEG-based precipitation (CP)^4,17^, and iZON column-based size exclusion chromatography (SEC) column^3^, complements a thorough investigation of our method (Figs. 4 and 5, Supplementary Table S1). Further, we evaluated the relevance of our method in downstream applications like multiple omics experiments. This comparative analysis scrutinizes not only the yield but also the purity of the isolated sEVs, providing valuable insights into the efficacy of our protocol in comparison to established methods in the field. This research thus contributes to the refinement of sEV isolation techniques, fostering advancements in studying these extracellular vesicles across various biological fluids. All methods were performed in accordance with the relevant guidelines and regulations.

### Biological sample collection & processing

 All India Institute of Medical Sciences’ institutional ethics committee in New Delhi, India, provided the ethical approval. This study has been given ethical approval under the number IEC-281/07.05.2021, dated 08-05-2021. The methodologies used in this study aligned with the standards outlined in the Declaration of Helsinki. Once the written consent form was obtained, all of the subjects (healthy controls, *N* = 10) were enrolled in the study. In exchange for their participation in this study, the subjects were given a comprehensive written participant information sheet (PIS) and participant informed consent form (PICF), which they signed. 5–5 ml of unstimulated saliva and peripheral blood were collected from healthy individuals. SH-SY5Y neuroblastoma cells were purchased from NCCS, Pune. For the collection of saliva, plasma, and media samples for sEV isolation, the undermentioned protocol was followed (Figs. 4 and 5, Supplementary Table S1)^14–16^. For saliva, 5 ml of unstimulated saliva sample was collected from the bottom of the mouth while the subject was at rest in a 50 ml sterile centrifuge tube placed on ice. To eliminate debris as a pellet, the saliva samples were centrifuged for 20 min at 4 °C at 1,800 g. The supernatant was then aliquoted into sterile microcentrifuge tubes (MCT). For plasma, 5 ml of peripheral blood was drawn via venepuncture in EDTA (anticoagulant) vials (BD Biosciences) from healthy subjects following “WHO Guidelines on Drawing Blood: Best Practices in Phlebotomy, 2010”^30^. The blood samples were then centrifuged at 2,500 g for 15 min at 4 °C to separate the plasma. Once the plasma (supernatant) was separated on top and collected; RBC, WBC, and platelets were pelleted down into the tube’s bottom. For media, 10 ml of conditioned F12 media (Gibco, 21700-075) was collected from a 95% confluent T-25 cell culture flask into a sterile centrifuge tube after the SH-SY5Y neuroblastoma cells (sourced from the National Center for Cell Science, Pune) were removed. The media was subjected to serial centrifugation at 300 g for 10 min at 4 °C to remove the dead cell, and the supernatant was collected into a separate new tube. To clarify the sample, another centrifugation step at 2,500 g for 20 min at 4 °C was performed, and the supernatant was collected into a new tube. Furthermore, separated supernatants from respective samples were transferred into a sterile MCT followed by a centrifugation step at 10,000 g for 30 min at 4 °C, and the supernatants (clarified samples) were collected. For immediate use in further investigations, the clarified samples were stored at 4 °C or on ice; for long-term storage, they were stored at -80 °C. To generate a clear supernatant for additional processing, the clarified samples that had been held at -80 °C were first thawed on ice for two hours before usage, followed by an additional centrifugation step at 10,000 g for 30 min at 4 °C, and the supernatants (clarified samples) were processed for sEV isolation.

## Isolation of sEVs

We used four different methods for sEVs isolation from the three types of samples: PEG-based precipitation (CP), PEG-based precipitation combined with ultrafiltration (CPF), ultracentrifugation (UC), and Size Exclusion Columns (SEC). The protocols used for these four methods are described as follows (Fig. 5e – i, Supplementary Table S1).

### PEG-based precipitation (CP - Method 1)

The sEV were isolated from clarified samples (saliva, plasma, cell culture media) through PEG-based precipitation^14^. The detailed protocol is as follows (Fig. 5f). To eliminate bigger vesicles and particles, the cleared samples were run through a 0.22 μm membrane syringe filter. A 50% polyethylene glycol (PEG) 6000 solution in Milli-Q water was mixed with a filtered sample to obtain a final 14% PEG concentration in each sample in a new MCT. The starting volume of clarified saliva and media was 720 µl with 280 µl of 50% PEG-6000, and for plasma, it was 360 µl with 140 µl of 50% PEG-6000. Each tube was vortexed for 30 s to mix the solution thoroughly, followed by incubation for 8–10 h or overnight at 4 °C. Further, the next day, the incubated sample was taken out from 4 °C and centrifuged at 13,000 g for 1 h at 4 °C to pellet down the sEVs. Inverting and tapping the tube removed the supernatant from the MCT. The pellet was washed by adding 100 µl of 1× PBS and decanting the PBS solution. This process was repeated twice. After adding 100 µl of 1× PBS and decanting the PBS solution, the pellet was cleaned. This procedure was carried out twice. The sEV pellets from saliva and media samples were resuspended in 100 µl, and the pellet from the plasma sample was resuspended in 200 µl of 1× PBS for downstream analysis.

### PEG-based precipitation with ultrafiltration (CPF - Method 2)

 A high yield of sEV was obtained by the PEG-based precipitation approach, and homogeneity and purity were achieved by the two-step filtration procedure^15^. For this approach, the PEG-precipitation procedure described in CP-Method was used to separate the sEVs from the cleared samples, followed by ultrafiltration as described below (Fig. 5g). For the ultrafiltration process, we used the 100 kDa cut-off centrifugal filters. Initially, centrifugal filters were washed by adding 500 µl of 1× PBS and centrifuged at 10,000 g for 2–3 min at 4 °C in a fixed-angle rotor to wash and equilibrate the membrane of the filter. Furthermore, isolated sEVs from PEG-precipitation were diluted with 1× PBS in a 1:1 ratio (200 µl sEV suspension and 200 µl PBS) and loaded into the inner tube of the centrifugal filter. The sEV sample was centrifuged at 10,000 g until the filter’s volume was reduced to 100–150 µl at 4 °C. The sEVs were carefully taken out from the inner tube of the centrifugal filter using a pipette and used for downstream characterization.

### Ultracentrifugation (UC - Method 3)

The sEV were isolated from clarified samples (180 µl of clarified plasma, 360 µl of clarified saliva, and media samples) using ultracentrifugation, and the detailed protocol is as follows (Fig. 5h). The clarified samples were filtered using a 0.22 μm filter before progressing to ultracentrifugation. To pellet down sEVs, the samples were centrifuged at 120,000 g for 90 min at 4 °C in a Beckman Coulter Optima L-80XP ultracentrifuge equipped with a Type 50.2 Ti fixed angle rotor. After carefully removing the supernatant, the pellets containing sEV were resuspended in 1 ml of ice-cold PBS. A second round of centrifugation at 120,000 g for 90 min at 4 °C was done for the washing, and the resulting sEV pellet was resuspended in 100 µl of 1× PBS.

### Size exclusion chromatography columns (SEC - Method 4)

The clarified samples were processed, and sEVs were isolated using qEV columns according to the manufacturer’s protocol (Fig. 5i). The column and 1× PBS buffer were brought to room temperature and kept the column upright. After flushing the column with two column volumes (20 ml x 2) of 1× PBS buffer and removing the bottom cap, the buffer was able to pass through the column. 500 µl of the clarified sample was loaded into the column and allowed to run. The column stopped flowing when the samples entered the loading frit. The column was filled with 2 ml of 1× PBS, and four void elution fractions of 500 µl (fractions 01 to 04) were collected from the bottom cap of the column. Ten 500 µl elution fractions (fractions 05 to 14) were extracted from the column’s bottom cap after 5 ml of 1× PBS was poured into the top of the column. After extracting the necessary fractions, the column was washed and cleaned to remove any leftover proteins. To restore the pH to normal before adding another sample, the column was immediately cleaned with 10 ml of 0.5 M NaOH and 20 ml of 1× PBS upon completion of fraction collection. 10 ml of 1× PBS with 1% Sodium azide was added to the column and stored at 4 °C.

## sEV characterization

### Transmission Electron Microscopy (TEM)

 Using TEM, the ultrastructural morphology of isolated sEV from each protocol was examined^15^. The negative Staining method was used to observe sEVs under TEM, and the protocol is as follows. sEV samples were diluted (plasma 1:5000, saliva 1:1000, and media 1:250) with filtered 1× PBS. The diluted sEV suspension was filtered with 0.22 μm filters before further processing. sEV samples were placed on fresh parafilm in 50–100 µl drops. Similarly, filtered PBS or filtered double-distilled water and 2% aqueous uranyl acetate were placed on parafilm in 50–100 µl drops. sEV were allowed to get adsorb for 20–30 min at room temperature on a 300-mesh carbon-coated copper grid (Ted Pella, 01843). After blotting dry, the grids were rinsed with PBS or filtered double-distilled water for a minute at room temperature. After once more blotting drying, the grids were negatively stained for 15 to 20 s with a 2% aqueous uranyl acetate solution. Grids were then examined using a transmission electron microscope after being blot-dried.

### Nanoparticle tracking analysis (NTA)

It facilitates the measurement of both the nanoscale particle size and concentration. The Zeta View Twin system from Particle Metrix, Germany, was used to quantify sEV. In the scatter mode of the Zeta View Twin system, the concentration of sEV was measured in particle/ml^15^. The protocol for scatter-mode NTA for sEV quantification is as follows. For the NTA experiment, the measuring cell was first washed by injecting MilliQ water with a 1 ml syringe, followed by washing it with 1 ml of filtered 1× PBS or the same buffer that was used for sEV dilution. The samples were diluted with 1× PBS into a microcentrifuge tube in a 1:1000 ratio in all methods except SEC where a 1:100 ratio was used. Samples were vortexed for 30 s before loading into the NTA instrument. sEVs were loaded into the measuring cell by injecting diluted sEV suspension. Keep the syringe plugged in during the measurement to recover the sample afterward. Three cycles of scanning eleven cell places, each with 60 frames per position (video setting: high) were carried out using the following parameters: Focus: automatic focus, all sample camera sensitivity values are 80.0; shutter and scattering intensity are 150 and 5.0, respectively; 488 nm embedded laser; cell temperature is 25 °C. A CMOS camera was used to record the videos, which were then examined using the built-in ZetaView Software 8.05.12 with the following analysis parameters: maximum particle size: 1000; minimum particle size: 10; minimum particle brightness: 30.

### Bicinchoninic acid (BCA) method

For protein Quantification, bovine serum albumin (BSA) Stock (20 mg/ml) was prepared for making standard solutions of eight different concentrations (0, 125, 250, 500, 750, 1000, 1500, 2000 µg/ml). The Commercial (BCA) reagents were used. Combine reagents A and B in a 50:1 ratio to produce a working solution. 200 µl working BCA reagents were added to each well of a 96-well plate. 10 µl of sample and BSA standard were added and incubated at 37ºC for 45 min. The absorbance reading was taken on a Spectramax spectrophotometer at 562 nm and was plotted on a graph to ascertain the protein concentrations in the samples.

### SDS PAGE and western blotting

To evaluate the profile of common exosomal markers (CD63, CD9, Flotillin-1, and TSG101), Western blots were performed as follows. In SDS-PAGE, 2 µg protein of saliva, 5 µg protein of plasma, and 15 µg protein of media-derived sEVs were loaded with the 5× SDS sample loading dye. The sample was heated at 95 °C for 10 min, followed by a spin on 10,000 g for 1 min. The supernatant was carefully removed and loaded on the 12% SDS PAGE. After running the SDS PAGE, the gel was removed from the SDS-PAGE cassette and put into Milli-Q water for rinsing. The obtained gel was subjected to wet-mode western blotting using the BioRad western blotting apparatus. The proteins were transferred from the gel to 0.22 μm PVDF membrane (80 V for 1 h). After transferring, the blot was removed and washed with 1X TBS, followed by blocking using 3% BSA in Tris-base saline containing 0.1% of Tween 20 (TBST). The blot was washed by adding TBST buffer and kept on gentle rocking for 5 min. This step was performed thrice. The blot was incubated with the primary antibody (1:5000 dilution of antibody in 1.5% BSA in TBST) anti-CD63 (10628D, Invitrogen), anti-Flotillin-1 (PA5-17127, Invitrogen), anti-CD9 (PA5-86534), anti-TSG101 (Invitrogen, MA5-32462), anti-Apolipoprotein B antibody (MA5-14671, Invitrogen), and anti-β-actin antibodies at 4 °C overnight. The next day, the primary antibody was removed from the blots and washed by adding TBST buffer and kept on gentle rocking for 5 min. This step was performed thrice. A subsequent secondary antibody was added to the blot in a 1:10000 dilution and incubated for 2 h at room temperature on a rocker. The blot was again washed with 1XTBST 3 times. Finally, blots were developed using HRP-based electroluminescence using the Femto LUCENT™ PLUS-HRP kit (Gbiosciences, AD0023).

### Laser confocal microscopy

 The purity of sEVs of the CPF method (our) was further evaluated with laser confocal microscopy with an anti-CD9 antibody, and the protocol is as follows. Three groups of sEVs were used during this experiment: sEVs isolated with the CP method, sEVs isolated with the CP method followed by filtration with a 0.22 μm syringe filter, and sEVs isolated with the CPF method. 10 µl sEVs suspension from each group (in 1× PBS) was taken in sterile amber-colored MCTs. The sEV suspension was incubated with 0.5 µg of anti-CD9 Alexa Fluor 488-conjugated antibody (R&D Systems, FAB1880G) for 2 h at 25℃ in the dark. 5 µl of antibody incubated sEV suspension was mounted onto a glass slide with a glass coverslip and immediately observed under the Zeiss LSM980 laser confocal system.

## Downstream processing of sEVs for OMICS analysis

To effectively lyse the plasma-derived sEVs isolated using the CPF technique, they were subjected to freeze-thaw cycles and then ultrasonication using an amplitude of 25 for two minutes, with 30-second on/off cycles. After centrifuging them for 10 min at 10,000 g, the resulting supernatant was used in further investigations. The sEVs underwent downstream processing for small RNA sequencing (miRNA) studies and liquid chromatography with tandem mass spectrometry (LC-MS/MS) (proteomics and lipidomics).

### Processing of sEVs proteomics analysis

 High-select Depletion Spin Columns (Thermo Scientific, A36366) were utilized to remove highly abundant proteins from the plasma before isolating sEVs. After sEVs isolation, the BCA technique was used to prepare the sample in accordance with the manufacturer’s instructions and measure the total protein concentration of plasma-derived sEVs; 100 µg of total protein was used in further procedures. After gently vortexing to combine the 5 mM dithiothreitol (DTT), the mixture was incubated for 30 min at 37 °C. After gently vortexing with 15 mM iodoacetamide (IAA), the mixture was incubated in the dark for 30 min at room temperature (25 °C). A 1:50 ratio (trypsin: protein) was obtained by adding 2 µl of trypsin (1 µg/µl). For protein digestion, samples were stored at 37 °C for the entire night. 0.1% formic acid was added to halt the enzymatic process, and the pH was carefully kept between 3.0 and 4.0. ZipTip was used to desalt the tryptic peptides using C18 resin (Millipore, ZTC18S096). The samples underwent vacuum drying and reconstitution in 0.1% (v/v) formic acid following elution prior to LC-MS/MS analysis. The samples were analyzed using Orbitrap LC-MS/MS (Thermo Scientific).

### Processing for sEV MiRNA sequencing analysis

200 µl of sEVs in 1× PBS was utilized as the input volume for RNA extraction. Total RNA was extracted from sEVs using a Total RNA Purification kit (Norgen Biotek, 17200), according to the manufacturer’s instructions. Using the QIAseq miRNA Library Kit procedure, small RNA sequencing (smRNA) libraries were created (Qiagen, 331502). The Qubit fluorometer (Thermo Scientific) was used to quantify the Illumina-compatible sequencing libraries, and the Agilent 2200 TapeStation was used to analyze the fragment size distribution of the libraries. The sequencing was carried out using the Illumina NovaSeq 6000 sequencing platform in accordance with the manufacturer’s guidelines.

### Processing for sEV lipidomics analysis

 To isolate total lipid from sEVs, a solvent mixture having chloroform, methanol, and MilliQ water in a ratio of 8:4:3 was used, following the Folch method. 300 µl of ice-cold solvent was added to 15 µl of sEV suspension. The solution was vortexed vigorously for 5 min to separate the organic and aqueous layers. The organic layer (bottom layer) was pipetted out carefully without disturbing the aqueous layer. 80 µl of chloroform was added to the residual layer and vortexed for 5 min. The remaining organic layer was pipetted out carefully and mixed with the already collected one. This collected organic layer contains total lipids isolated from sEV suspension and was further processed for Orbitrap LC-MS/MS (Thermo Scientific) analysis.

### Statistical analysis

GraphPad Prism 8.0 was used to analyze the descriptive statistical analysis for all experiments used for the characterization of isolated sEV, nanoparticle tracking analysis, and densitometric analysis. The unpaired student t-test was performed to ascertain the statistical significance. A significance threshold of *p* < 0.05 was applied. We discovered throughout the statistical analysis that the data did not fit into a normal distribution because of a variety of reasons, including small sample sizes and data outliers. For this reason, we have chosen non-parametric testing. For three or more data groups, we have included the median value along with a post hoc Dunn and a non-parametric Kruskal-Wallis test. There are no one-sided statistical tests employed.

## Electronic supplementary material

Below is the link to the electronic supplementary material.

Supplementary Material 1

## Data Availability

The data set that supports the current study is available from the corresponding author upon reasonable request. The mass spectrometry proteomics data have been deposited to the ProteomeXchange Consortium via the PRIDE partner repository with the dataset identifier PXD057253.
