# Supplementary material for: A simplified and efficient method for isolating small extracellular vesicles for comparative and comprehensive translational research
Source: Sci Rep. 2025 May 11;15:16367. doi: 10.1038/s41598-025-99822-y (PMC12066714; doi:10.1038/s41598-025-99822-y)
Supplement: Supplementary file 1 — Supplementary Material 1 [file 41598_2025_99822_MOESM1_ESM.docx]

**SUPPLEMENTARY INFORMATION**

**Supplementary Table S1: List of precautions for performing related procedures during sEVs isolation and characterization.**

| **Procedure** | **Precautions** |
| --- | --- |
| Saliva Collection and Processing | - The subject/patient should have maintained all oral hygiene during the sample collection. They should not have taken any meal one hour before the sample collection. They should have brushed their teeth or gargled with mouthwash before sample collection. - Clarified saliva samples stored at -80°C should be aliquoted into 500µl aliquots, sufficient for the downstream analysis of sEVs. - Sputum samples should not be considered saliva samples. Initial centrifugation will help differentiate viscous sputum from saliva. |
| Plasma Collection and Processing | - Blood samples should be kept at room temperature before centrifugation to check for any hemolysis in the sample. - One has to be very careful when pipetting out the clarified plasma from the pelleted RBC and WBC. |
| Media Collection and Processing | - Media should not have a high percentage of dead cells. - For better sEV yields in the SH-SY5Y cell line, the media should be collected after 24 hours of splitting the cells. - If other cell lines are used for sEV isolation, the time point to collect conditioned media should be optimized. - The media should be collected at 4-5 passages after revival of the cells for better sEV yields. |
|  | - Saliva is a viscous fluid; therefore, it is recommended to dilute it by adding 1× phosphate buffer saline (PBS) in a 1:1 ratio before filtering through a 0.22µm membrane syringe filter. - PBS should be ice-cold and filtered with a 0.22µm membrane syringe filter before use. - Filtration of the clarified samples using a 0.22µm membrane syringe filter is recommended; however, this is optional due to the sample loss. - While resuspending the pellet, minimum numbers of pipetting strokes should be optimized (e.g., 10 strokes) |
| PEG-based Precipitation (CP - Method 1) | - A freshly prepared 50% PEG solution was used to precipitate sEVs. The solution was prepared by dissolving the 50 grams of PEG-6000 into 100 ml of autoclaved MilliQ water. The solution was mixed with the help of vortexing. - The washing step is necessary to remove PEG contamination from the sEVs. - sEV isolation should be performed using an equal volume of fluid from each sample to maintain homogeneity during the experiment. |
| PEG-based Precipitation with Ultrafiltration (CPF - Method 2) | - During centrifugation at step 3, the reduced volume should be observed to check the required volume. |
| Ultracentrifugation (UC - Method 3) | - The sEV pellet from ultracentrifugation has a slimy consistency; therefore, be cautious during pipetting. |
| Size Exclusion Chromatography Columns (SEC - Method 4) | - At any time, the column should not be dried out. - Always use a filtered buffer. - Wait until the column reaches operating temperature before removing the column lids. - Stopping the column flow should be avoided during the run to ensure accurate EV separation. - To ensure sEV isolation, all the fractions should be analyzed with NTA or protein estimation. |
| Transmission Electron Microscopy | - Use only non-magnetic type tweezers to hold grids. - If the prepared grids are not viewed on the same day, sEV’s fixation with glutaraldehyde or paraformaldehyde should be used. - The dilution range should be optimized. - The staining time and dilution should be optimized. - The grids should be blot-dried after each step. - The coated side should be touched during blot drying or handling. |
| Nanoparticle Tracking Analysis | - All the buffer and water used in the NTA experiment should be freshly prepared and filtered through a 0.22 µm syringe filter. - NTA instrument should be calibrated by injecting the polystyrene beads of known size - After the measurement has been carried out, the sample from the NTA experiment can be recovered by drawing the sEV sample back into the syringe. However, this may cause bubbles to form, so precautions should be taken. |
| SDS PAGE and Western Blotting | - If using a PVDF membrane for western blotting, activate it by soaking it in a 100% methanol solution for five minutes and then rinsing it with 1X TBS. |
| Processing of sEVs Proteomics Analysis | - The plasma/serum samples should be depleted for the highly abundant proteins for proteomics. - To stop the trypsin digestion, the temperature variation method can also be used by transferring the samples to 4°C and incubating them for 30 minutes. - C18 resin columns can be used instead of ZipTip/C18 resin pipette tips for desalting. |
| Processing for sEV miRNA Sequencing Analysis | - The amounts of RNA in plasma-derived sEVs are usually low (picogram range), so it is not recommended to quantify RNA produced from sEVs using traditional techniques such as Nanodrop or fluorometer, which lack sufficient sensitivity [19]. - Another method to measure RNA is using a bioanalyzer regarding RIN (RNA integrity numbers), where RIN levels are determined by dividing the 28S rRNA by the 18S rRNA [20]. However, plasma-derived sEV has a deficit of rRNA (21–23), leading to a very low RIN value. |
| Processing for sEV Lipidomics Analysis | - For lipidomics, the remaining aqueous layer should also be run for an LC-MS/MS experiment, as all lipids could not be isolated from biofluids. |


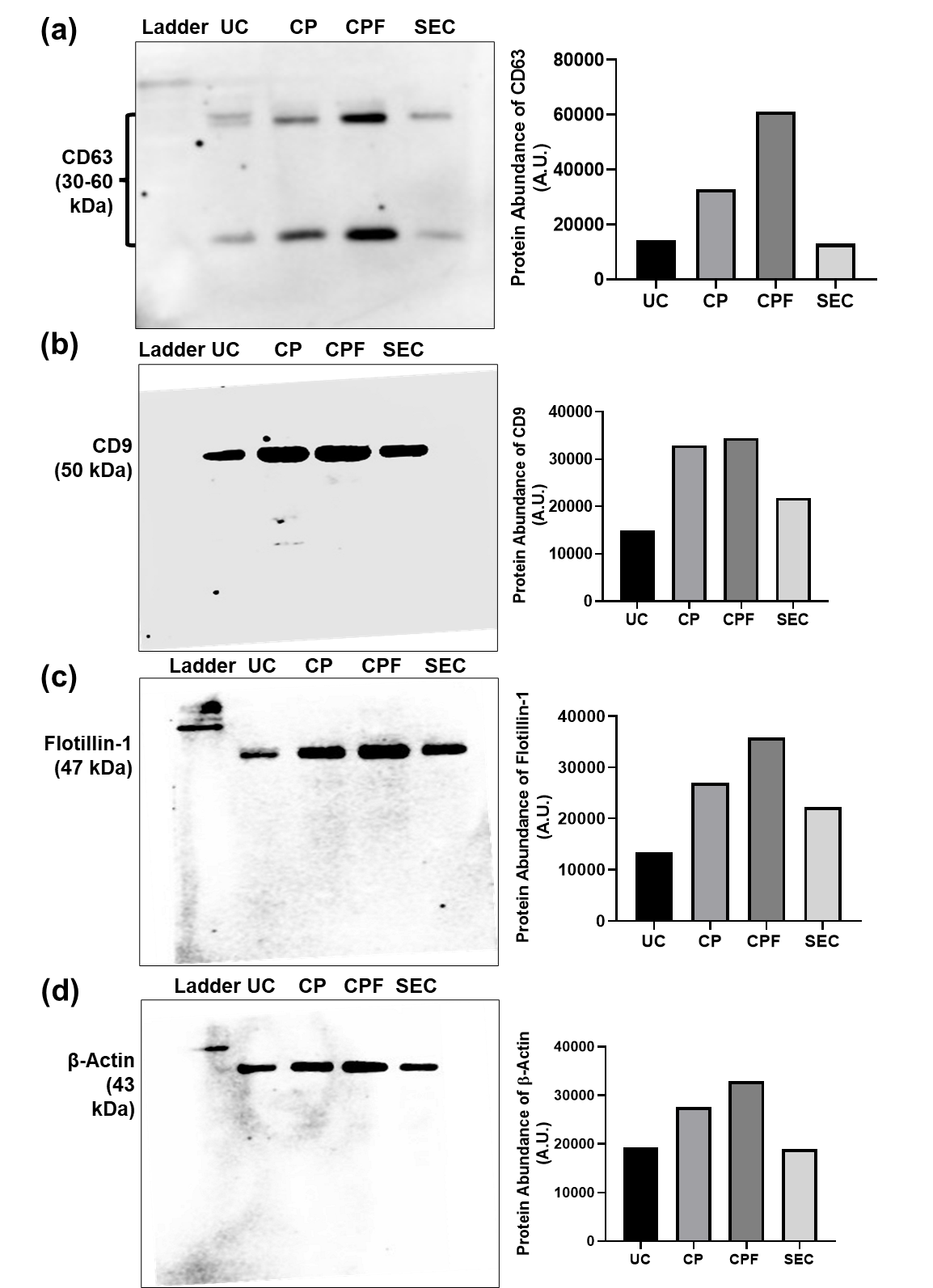


**Supplementary Figure S1:** **Expression profiles of salivary sEVs markers.** The Western blot of CD63 and its densitometric analysis (a), CD9 and its densitometric analysis (b), Flotillin-1 and its densitometric analysis (c), and β-actin (loading control) and its densitometric analysis (d). The sEVs isolation methods are precipitation (CP), precipitation with ultrafiltration (CPF), ultracentrifugation (UC), and size exclusion chromatography (SEC) columns.


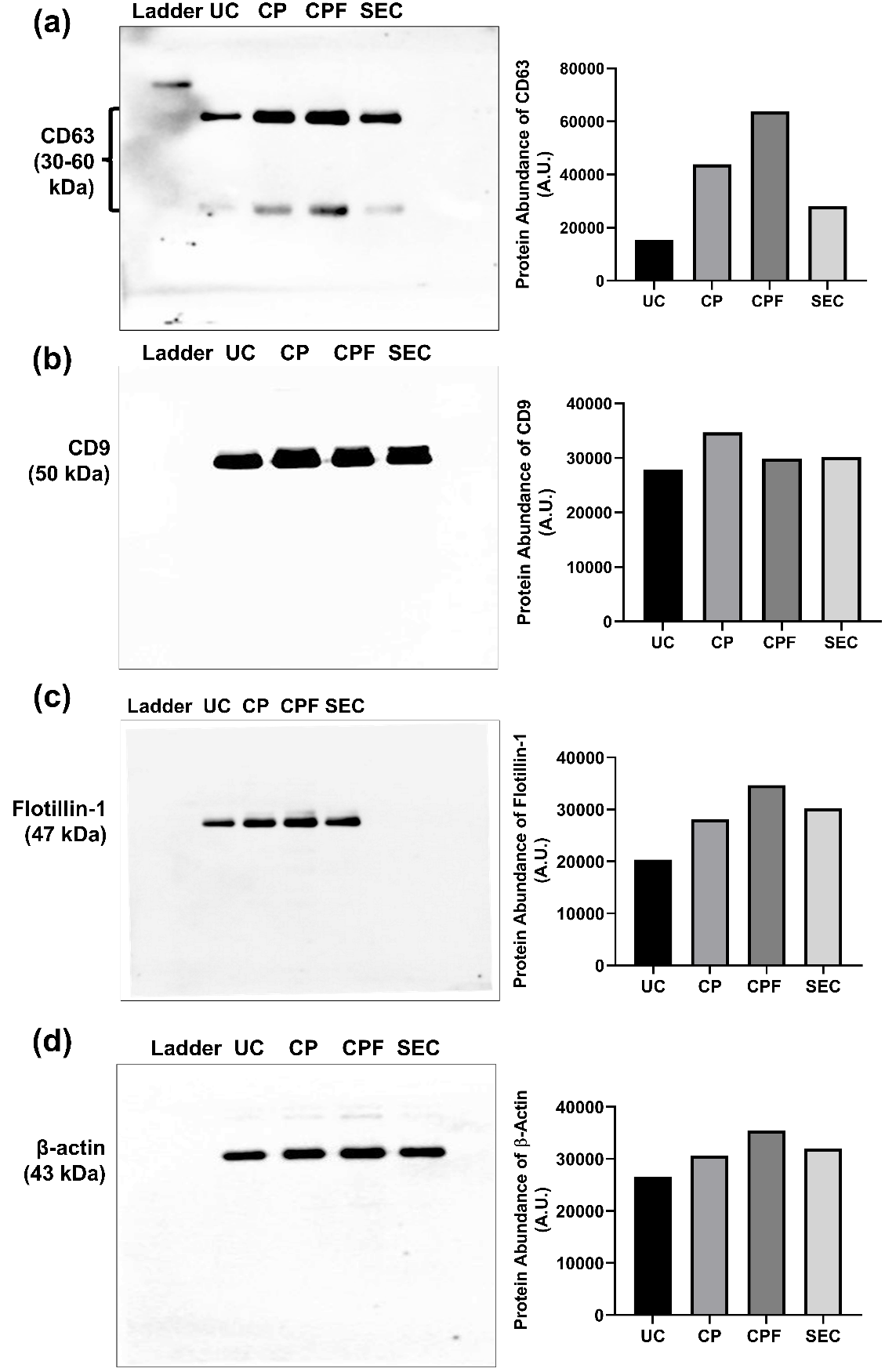


**Supplementary Figure S2:** **Expression profiles of plasma-derived sEVs markers.** The Western blot of CD63 and its densitometric analysis (a), CD9 and its densitometric analysis (b), Flotillin-1 and its densitometric analysis (c), and β-actin (loading control) and its densitometric analysis (d). The sEVs isolation methods are precipitation (CP), precipitation with ultrafiltration (CPF), ultracentrifugation (UC), and size exclusion chromatography (SEC) columns.


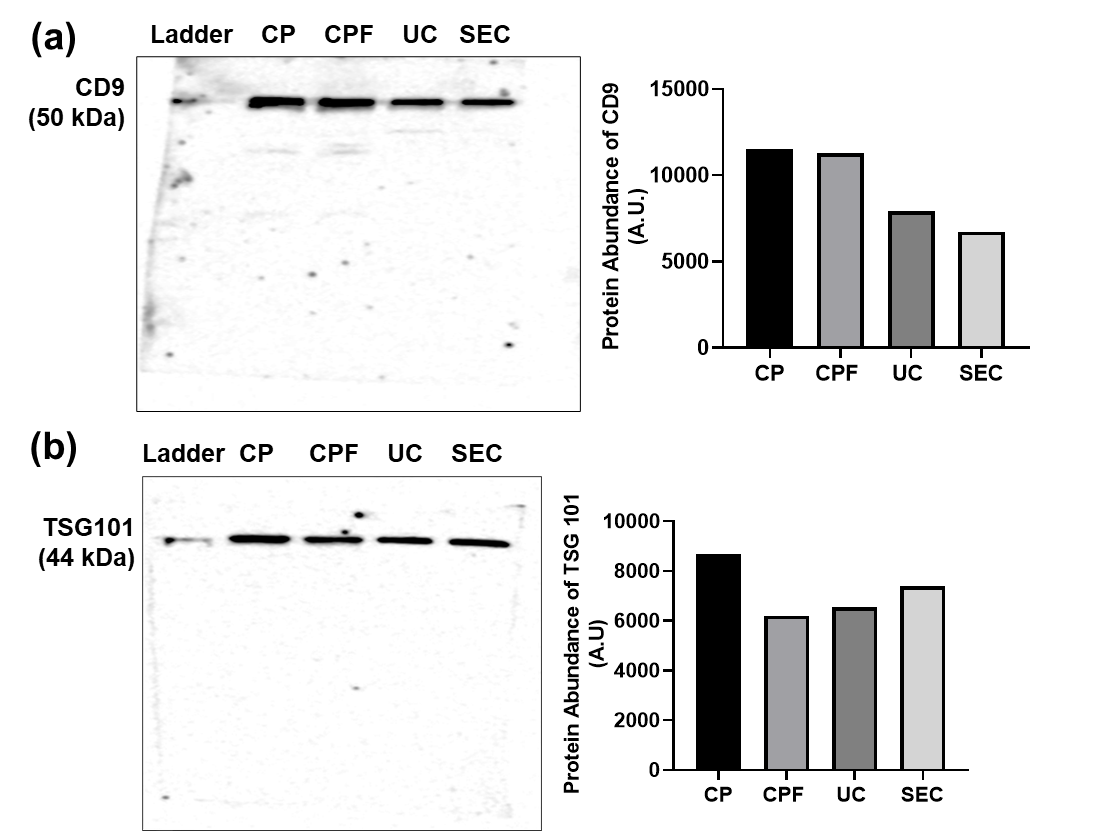


**Supplementary Figure S3:** **Expression profiles of conditioned cell culture media-derived sEVs markers.** The Western blot of CD9 and its densitometric analysis (a), and TSG101 and its densitometric analysis (b). The sEVs isolation methods are precipitation (CP), precipitation with ultrafiltration (CPF), ultracentrifugation (UC), and size exclusion chromatography (SEC) columns.


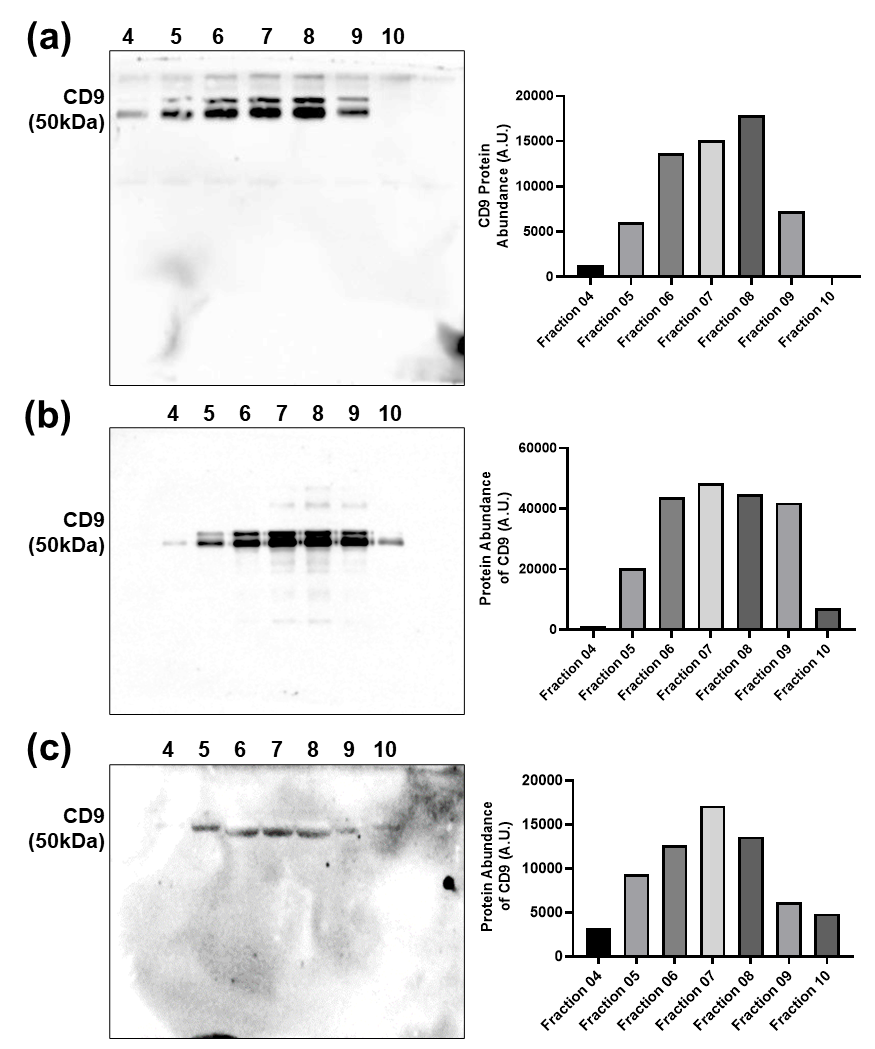


**Supplementary Figure S4:** **Expression profiles of CD9 in the SEC method isolated sEVs.** CD9 expressions in different fractions of sEVs isolated using the SEC method from saliva (a), plasma (b), and conditioned cell culture media (c) with their respective densitometric analyses.


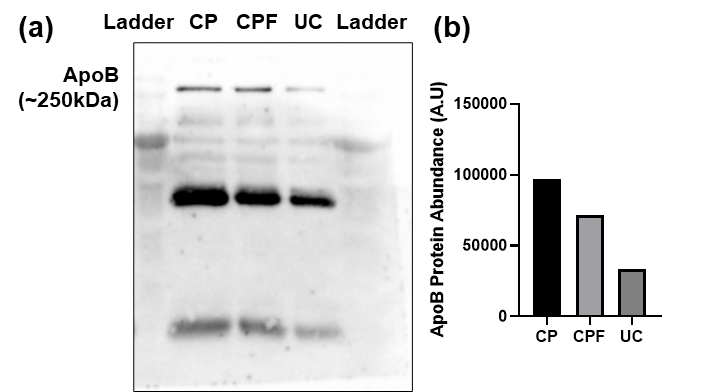


**Supplementary Figure S5:** **Expression profiles of ApoB in the sEVs isolated by CP, CPF, and UC methods.** ApoB expressions (4µg protein loaded in each sample) in sEVs isolated using different methods from plasma (a) with densitometric analyses (b).


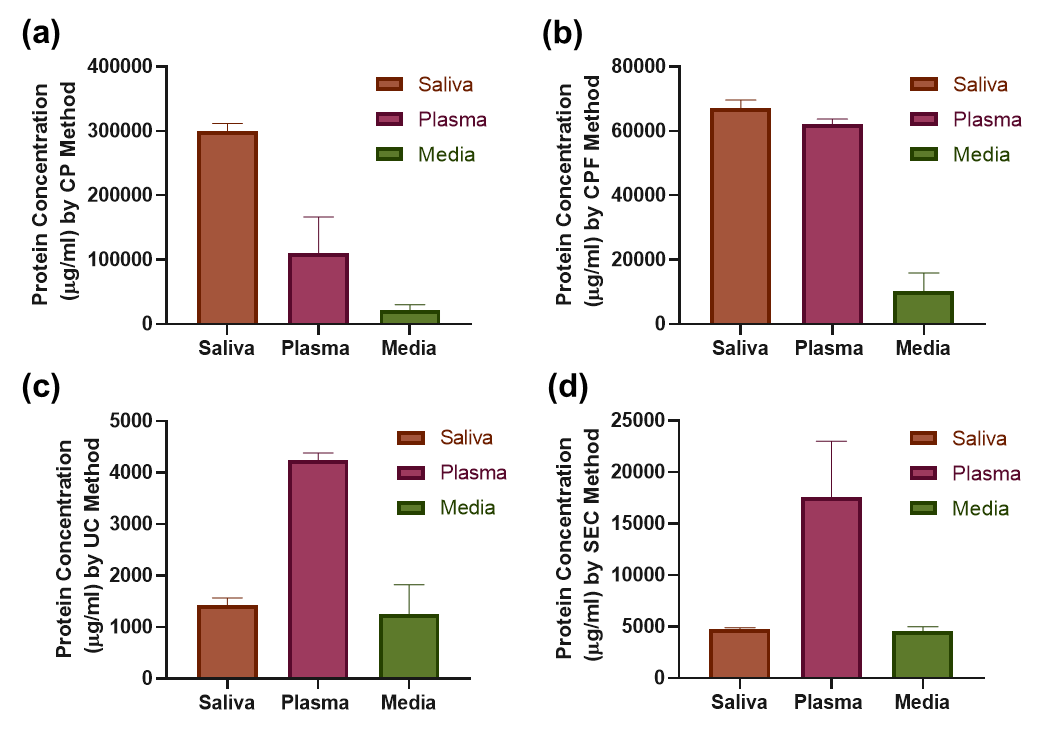


**Supplementary Figure S6:** Protein content in sEVs isolated by CP method (a), CPD method (b), UC method (c), and SEC method (d) from three sample types (Supplementary Figure 2b in the manuscript).


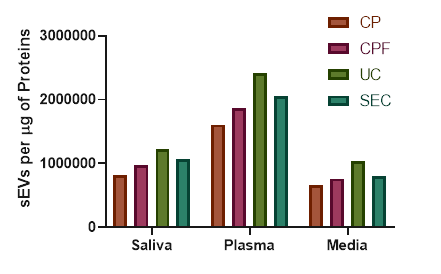


**Supplementary Figure S7:** Particle to protein ratio across different methods in three sample types.

**Supplementary Table S2: sEVs concentration and size distribution.**

| **Isolation Method** | **sEVs Concentration**  **(particle/ml) (Mean±SEM)** | | | **sEVs Size (nm) (Mean±SEM)** | | |
| --- | --- | --- | --- | --- | --- | --- |
|  | **Saliva** | **Plasma** | **Media** | **Saliva** | **Plasma** | **Media** |
| PEG-based Precipitation (CP) | 2.43E+11 ± 6.35E+10 | 1.76E+11 ± 4.25E+10 | 1.46E+10 ± 6.66E+08 | 81.87 ± 2.5 | 86.9 ±  3.9 | 76.13 ±  4.4 |
| PEG-based Precipitation with Ultrafiltration (CPF) | 6.5E+10 ± 2.08E+09 | 1.16E+11 ± 3.17E+10 | 7.83E+09 ± 1.60E+08 | 90.03 ± 3.8 | 94.13 ±  4.6 | 81.83 ±  4.8 |
| Ultracentrifugation (UC) | 1.74E+09 ± 2.90E+07 | 1.02E+10 ± 2.25E+09 | 1.3E+09 ± 1.52E+08 | 97.13 ± 4.3 | 97.83 ±  2.8 | 88.13 ±  5.1 |
| Size exclusion chromatography (SEC) column | 5.08E+09 ± 5.73E+08 | 3.6E+10 ± 8.82E+09 | 3.66E+09 ± 6.71E+08 | 95.5 ± 8.9 | 91.0 ±  8.8 | 84.97 ± 8.2 |

**List of Laboratory Reagents:**

1. Phosphate buffered saline (Himedia, ML116)
2. Antibiotic-Antimycotic (Gibco, 15240062)
3. Bovine serum albumin (Bio Basic, AD0023)
4. Ethyl alcohol (Merck, 100983)
5. EDTA (anticoagulant) vials (BD Biosciences, PC623601)
6. F12 media (Gibco, 21700-075)
7. 0.22µm syringe filter (Millipore, SLGPR33RS)
8. Polyethylene glycol (PEG) 6000 (Sigma, 81260)
9. Tris (Bio Basic, TB0194)
10. Glycine (Bio Basic, GB0235)
11. Sodium chloride (Qualigens, Q15915)
12. RIPA buffer (Sigma, R0278)
13. Sodium dodecyl sulphate (Sigma, 436143)
14. 100kDa cut-off centrifugal filters (Millipore, UFC-5100)
15. qEV original 70 nm columns (iZON, SP1)
16. Uranyl acetate (SRL Chemicals, 81405)
17. 300-mesh carbon-coated copper grid (Ted Pella, 01843)
18. Anti-CD63 antibody (Invitrogen, 10628D)
19. Anti-Flotillin-1 antibody (Invitrogen, PA5-17127)
20. Anti-CD9 antibody (Invitrogen, PA5-86534)
21. Anti-β-actin antibody (Invitrogen, MA1-140)
22. Anti-TSG101 antibody (Invitrogen, MA5-32462)
23. Anti-Apolipoprotein B antibody (Invitrogen, MA5-14671)
24. HRP-based electroluminescence using the Femto LUCENT™ PLUS-HRP kit (Gbiosciences, AD0023).
25. T-25 cell culture flask (Thermo Nunc, 156367)
26. T-75 cell culture flask (Thermo Nunc, 156499)
27. 50-ml conical centrifuge tube (Tarsons, 546041)
28. 15-ml conical centrifuge tube (Tarsons, 546021)
29. 1.5-ml centrifuge tube (Tarsons, 500010)

**List of Equipments:**

1. Microcentrifuge (Neuation)
2. Refrigerated Centrifuge (Dlab, D1524R)
3. Optima L-80XP ultracentrifuge (Beckman Coulter)
4. Type 50.2 Ti Fixed-Angle rotor (Beckman Coulter)
5. Spectramax i3x (Molecular Devices)
6. Weighing Balance (Radwag)
7. Bio-safety cabinet Class II, Type A2
8. Nanodrop One (Thermo Scientific)
9. 100-1000 µl pipette (Thermo Scientific)
10. 20-100 µl pipette (Thermo Scientific)
11. 0.5-10 µl pipette (Thermo Scientific)
12. 0.2-2 µl pipette (Thermo Scientific)
13. 1 ml syringe (BD Biosciences)
14. 5 ml syringe (BD Biosciences)
15. 0.22 syringe filter (Millipore)
16. Transmission electron microscope TALOS-S (HR-TEM) (Thermo Scientific)
17. Transmission electron microscope TECNAI G20 HR-TEM (FEI)
18. Zeta View Twin system (Particle Metrix, Germany)
